# Metagenomic Characterization of Gut Microbiota of Carriers of Extended-Spectrum Beta-Lactamase or Carbapenemase-Producing Enterobacteriaceae Following Treatment with Oral Antibiotics and Fecal Microbiota Transplantation: Results from a Multicenter Randomized Trial

**DOI:** 10.3390/microorganisms8060941

**Published:** 2020-06-23

**Authors:** Stefano Leo, Vladimir Lazarevic, Myriam Girard, Nadia Gaïa, Jacques Schrenzel, Victoire de Lastours, Bruno Fantin, Marc Bonten, Yehuda Carmeli, Emilie Rondinaud, Stephan Harbarth, Benedikt D. Huttner

**Affiliations:** 1 Genomic Research Laboratory, Division of Infectious Diseases, University Hospitals and University of Geneva, Rue Michel Servet 1, 1211 Geneva, Switzerland; vladimir.lazarevic@genomic.ch (V.L.); myriam.girard@genomic.ch (M.G.); nadia.gaia@genomic.ch (N.G.); jacques.schrenzel@hcuge.ch (J.S.); 2 Division of Infectious Diseases, Geneva University Hospitals and Faculty of Medicine, Rue Gabrielle-Perret-Gentil 4, 1211 Geneva, Switzerland; stephan.harbarth@hcuge.ch; 3 Division of Internal Medicine, Beaujon Hospital, APHP, Boulevard du Général Leclerc 100, 92110 Clichy, France; victoire.de-lastours@aphp.fr (V.d.L.); bruno.fantin@aphp.fr (B.F.); 4 IAME Research Group, UMR 1137, INSERM and University of Paris, Rue Henri Huchard 16, 75870 Paris, France; 5 Department of Medical Microbiology, University Medical Centre, Heidelberglaan 100, 3584 CX Utrecht, The Netherlands; m.j.m.bonten@umcutrecht.nl; 6 Julius Center for Health Sciences and Primary Care, Universiteitsweg 100, 3584 CG Utrecht, The Netherlands; 7 National Institute for Antibiotic Resistance and Infection Control, Tel Aviv Medical Center, and Sackler Faculty of Medicine, Tel Aviv University, Weizmann Street 6, Tel Aviv 6423906, Israel; yehudac@tlvmc.gov.il; 8 Department of Medical Microbiology, APHP, Bichat-Claude-Bernard Hospital, Rue Henri Huchard 46, 75018 Paris, France; emilie.rondinaud@aphp.fr; 9 Infection Control Program and WHO Collaborating Center, Geneva University Hospitals, Rue Gabrielle-Perret-Gentil 4, 1211 Geneva, Switzerland

**Keywords:** fecal microbiota transplantation, extended-spectrum beta-lactamase-producing Enterobacteriaceae, carbapenemase-producing Enterobacteriaceae, microbiome, whole metagenome shotgun sequencing

## Abstract

**Background:** The R-GNOSIS (Resistance in Gram-Negative Organisms: Studying Intervention Strategies) WP3 study was the first multicenter randomized clinical trial systematically investigating fecal microbiota transplantation (FMT) for intestinal decolonization of extended-spectrum beta-lactamase-producing Enterobacteriaceae (ESBL-E) or carbapenemase-producing Enterobacteriaceae (CPE). Here, we characterized the temporal dynamics of fecal microbiota changes in a sub-cohort of the R-GNOSIS WP3 participants before and after antibiotics/FMT using whole metagenome shotgun sequencing. **Methods:** We sequenced fecal DNA obtained from 16 ESBL-E/CPE carriers having received oral colistin/neomycin followed by FMT and their corresponding seven donors. Ten treatment-naïve controls from the same trial were included. Fecal samples were collected at baseline (V0), after antibiotics but before FMT (V2) and three times after FMT (V3, V4 and V5). **Results:** Antibiotic treatment transiently decreased species richness and diversity and increased the abundance of antibiotic resistance determinants (ARDs). *Bifidobacterium* species, together with butyrate- and propionate-producing species from Lachnospiraceae and Ruminococcaceae families were significantly enriched in post-FMT microbiota of treated carriers. After FMT, the proportion of Enterobacteriaceae was lower compared to baseline but without statistical significance. **Conclusions:** Combined antibiotic and FMT treatment resulted in enrichment of species that are likely to limit the gut colonization by ESBL-E/CPE.

## 1. Introduction

Multidrug-resistant Gram-negative bacteria, such as extended-spectrum beta-lactamase-producing Enterobacteriaceae (ESBL-E) and carbapenemase-producing Enterobacteriaceae (CPE) were classified by the World Health Organization in 2018 as “critical” priority pathogens for research and development of new antibiotics [1]. This classification is justified by the significant burden of infections caused by these pathogens in terms of morbidity and mortality [2,3]. Since colonization precedes infection in most patients, there have been numerous attempts to eradicate carriage, albeit with little lasting success [4,5]. Indeed, the 2019 clinical guidelines of ESCMID-EUCIC (European Society of Clinical Microbiology and Infectious Diseases—European Committee on Infection Control) on decolonization of multidrug-resistant Gram-negative bacteria carriers do not recommend routine use of interventions aimed at achieving decolonization based on a systematic review and appraisal of the published literature [6]. 

Fecal microbiota transplantation (FMT) has gained increasing interest as intervention for decolonization of ESBL-E/CPE carriers due to its high efficiency and safety for the treatment of recurrent *Clostridioides difficile* infection. Furthermore, some animal experiments have shown promising results and numerous case reports and case series reporting anecdotal “success” of FMT for ESBL-E/CPE decolonization have been published over the last few years [7,8,9,10].

The recently published R-GNOSIS (Resistance in Gram-Negative Organisms: Studying Intervention Strategies) WP3 study [11] was the first randomized clinical trial assessing FMT for decolonization of ESBL-E/CPE carriers. While the intervention effect did not reach statistical significance (odd ratios for decolonization was 1.7 [95% CI 0.4–6.4]), possibly because of the failure to achieve the planned sample size, the loss of ESBL-E/CPE carriage detectable by culture was more frequent in FMT-treated participants than in the treatment-naïve control group. 

A better understanding of the dynamics of microbiota changes induced by antibiotic treatment and FMT, and of the role of donor microbiota would be crucial in selecting patients and donors for decolonization strategies. In this nested cohort study, we selected carriers and donors from the R-GNOSIS WP3 trial to explore these questions through metagenomic analyses of the collected stool samples.

## 2. Materials and Methods 

### 2.1. Description of Study Sub-cohort

Criteria for selection of carriers and donors into the R-GNOSIS WP3 study are explained in detail elsewhere [11]. Briefly, immunocompetent adults colonized with ESBL-E were eligible if they had experienced ≥1 episode of symptomatic infection with ESBL-E within ≤180 days before inclusion. Adults colonized with CPE did not have to meet this requirement. Thirty-nine patients in four centers (Geneva [Ge], Switzerland; Paris [Pa], France; Utrecht [Ut], the Netherlands; Tel Aviv [TA], Israel) were randomized to either control (no intervention; “treatment-naïve”) or a 5-day course of oral antibiotics (colistin and neomycin sulphate) followed by frozen FMT (named “FMT-treated” hereafter) from unrelated healthy donors. FMT was administered via oral capsules (Ge, Pa) or via nasogastric tube (Ut, TA). 

We selected a convenience sub-cohort of 26 ESBL-E/CPE carriers (Ge *n* = 12, Pa *n* = 7, Ut *n* = 5, TA *n* = 2) for the metagenomic analysis. Carriers were selected based on the fact that most of them had completed the last follow-up examination (V5) at the time when the official study/financing period ended (spring 2017); they thus represent the first individuals recruited for the main study. Twenty-four of 26 patients were colonized by ESBL-E and 8 were CPE colonized (of these 6 were also ESBL-E carriers). 

Of 26 patients, 16 were FMT-treated and 10 were treatment-naïve. We also selected 7 corresponding stool donors. The study was approved by local institutional review boards and national regulatory agencies in all centers, and all participants (recipients and donors) provided written informed consent.

The sex ratio (female:male) was 1 and the mean age was 63–64 years old in both FMT-treated and treatment-naïve groups (Table S1). The mean body mass index (BMI) of treatment-naïve and FMT-treated individuals was 31.7 and 26.2 kg/m^2^, respectively (Table S1); this difference was not associated with statistical significance (*t*-test, *p* = 0.054).

Stools were sampled from all carriers at five time points: baseline (V0), 8–14 days after randomization (V2) (after antibiotics but before FMT in the intervention group), 15–28 days after randomization (V3), 35–48 days after randomization (V4), and 5–7 months after randomization (V5). Details of the antibiotic treatment, FMT procedure and sample storage are reported in Supplementary Materials.

### 2.2. Whole Metagenome Shotgun Sequencing and Data Analyses

A detailed description of sample processing and metagenomic analysis is provided in Supplementary Materials. In total, we sequenced 21 samples from 7 donors, 76 from the 16 FMT-treated carriers and 47 from the 10 treatment-naïve carriers, with a mean number of raw read pairs of 5.5 M per sample. For donor microbiota, we sequenced DNA from aliquots of the native stools and/or FMT preparation, but for the purpose of this study, we analyzed only 12 stool suspensions used for FMT. Whole metagenome shotgun sequencing (2 × 150) was performed on a NextSeq 500 system (Illumina, San Diego, CA, USA).

Raw reads were quality-filtered with Trimmomatic v0.36 [12] and then mapped by Kraken2 [13] to a human genome (GRCh38.p7). Human-classified reads were removed and remaining sequences (on average 3.9 M read pairs per sample) were assigned to bacteria, viruses and fungi by Kraken2 using a confidence score of 80%. After the filtering steps, we identified 402 bacterial species belonging to the major gut microbiota phyla Firmicutes, Bacteroidetes, Proteobacteria and Actinobacteria. Moreover, host-depleted R1 reads were mapped against EzBioCloud 16S rRNA gene sequence [14] and ResFinder [15] databases with USEARCH v10 [16]. Statistical analyses were performed in PRIMER v7 (PRIMER-E Ltd., Plymouth, UK) and in the R software v3.2.3. 

## 3. Results

### 3.1. Donor Microbiota

**Figure 1:**
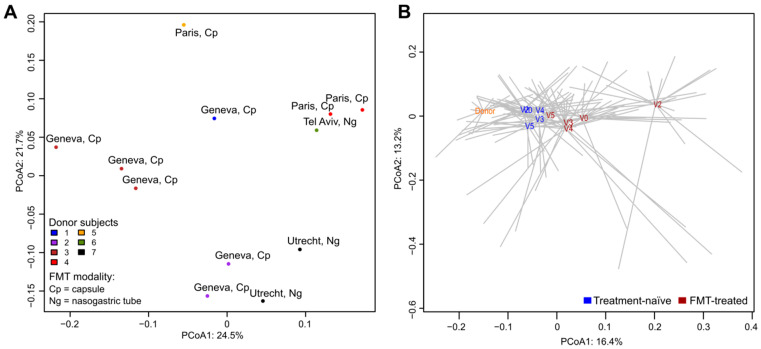
Global differences between microbial communities of fecal samples assessed by principal coordinates analysis (PCoA). The analyses were based on the relative abundance of bacterial species. The percentage of total data variance is reported for PCoA1 and PCoA2. (**A**) Samples from different donors. Data points are color-coded according to the donor. Each sample label reports the center of transplantation and the modality of FMT administration. (**B**) Comparison of samples from donors and FMT-treated and treatment-naïve carriers. Centroids (averaged microbiota profiles) of donors (orange) and of each sampling point (V0, V2, V3, V4 and V5) of treatment-naïve (blue) and FMT-treated (dark red) carriers are represented.

Since multiple samples from the same donor have been sequenced, we first looked at how the donor samples are related to each other. We found that samples from the same donor (administered to different carriers) clustered together in the principal coordinate analysis (PCoA) plot (Figure 1A). Donor samples also clustered according to the transplantation center and microbiota composition differed between the two FMT preparations (capsule and suspension for nasogastric administration) although not significantly (PERMANOVA, *p* = 0.06).

We then compared samples from donors with those collected from all time points of FMT-treated and treatment-naïve carriers. In the PCoA plot, donor samples clustered apart from other samples (Figure 1B) and they were significantly different in microbiota composition from FMT-treated and treatment-naïve microbiota (PERMANOVA, *p* < 0.05).

### 3.2. Impact of Antibiotic Treatment on Microbiota Profiles and Resistome of FMT-treated Carriers

**Figure 2:**
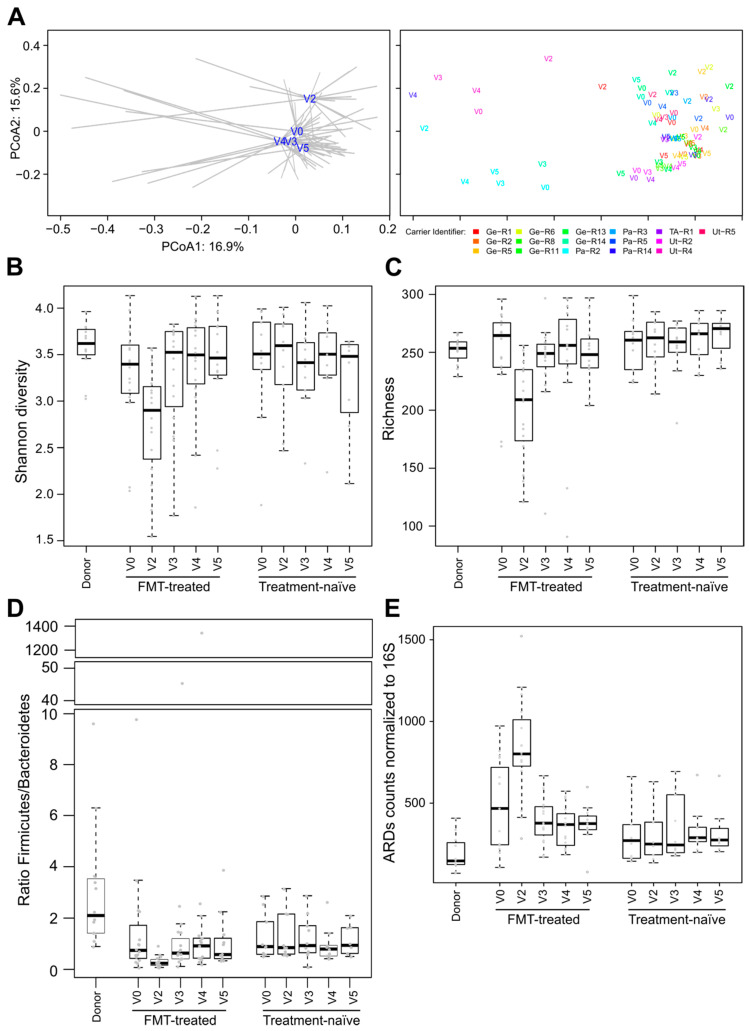
Effect of colistin/neomycin on microbiota composition of FMT-treated carriers. (**A**) PCoA plots of microbiota profiles computed on the species relative abundance of FMT-treated individuals. On the left, centroids (averaged microbiota profiles) from each time point of FMT-treated carriers are represented. Each sample is connected to its corresponding centroid with grey lines. On the right, samples are labeled according to the time point and colored according to the carrier. Shannon diversity (**B**) and species richness (**C**) were computed after rarefying sequencing reads to 40,000. (**D**) Ratio of Firmicutes to Bacteroidetes. (**E**) Number of reads assigned to ARDs and normalized to 1000 16S rRNA gene read counts.

Colistin/neomycin treatment had a significant effect on global microbiota composition. Microbiota at V2 (i.e., after antibiotic treatment but before FMT) was significantly different from microbiota of baseline (V0) and from later time points (PERMANOVA test, *p* < 0.05; Figure 2A). The administration of the study antibiotics caused a significant decrease of species diversity and richness (Wilcoxon signed rank test, *p* < 0.05; Figure 2B,C). 

At V2, we also observed a significantly decreased Firmicutes/Bacteroidetes ratio and a significant increase in the abundance of antibiotics resistance determinants (ARDs) compared to baseline (Wilcoxon signed rank test, *p* < 0.05; Figure 2D,E). In particular, the abundance of genes for tetracycline, aminoglycoside and beta-lactam resistance, which were the most detected ARDs, significantly increased at V2 compared to V0 (Wilcoxon signed rank test, *p* < 0.05, Figure S1). Importantly, we did not detect acquired ARDs to colistin (*mcr-1* to *mcr-5*), by querying the ResFinder database.

### 3.3. Post-FMT Microbiota and Its Association with Donor Microbiota

Decrease in species diversity and richness at V2 was followed by a significant increase of those indices at post-FMT time point V3 (Wilcoxon signed rank test, *p* < 0.05; Figure 2B,C). Likewise, after FMT administration, the Firmicutes/Bacteroidetes ratio and the ARDs content significantly increased and decreased, respectively (Wilcoxon signed rank test, *p* < 0.05), reverting to baseline levels (Figure 2D,E).

Post-treatment microbiota was similar to that of the baseline in terms of global species composition, richness and diversity (Figure 2). This was confirmed by the PERMANOVA test that revealed no statistically significant differences (*p* > 0.05) between V0 and post-FMT time points V3 and V4.

**Figure 3:**
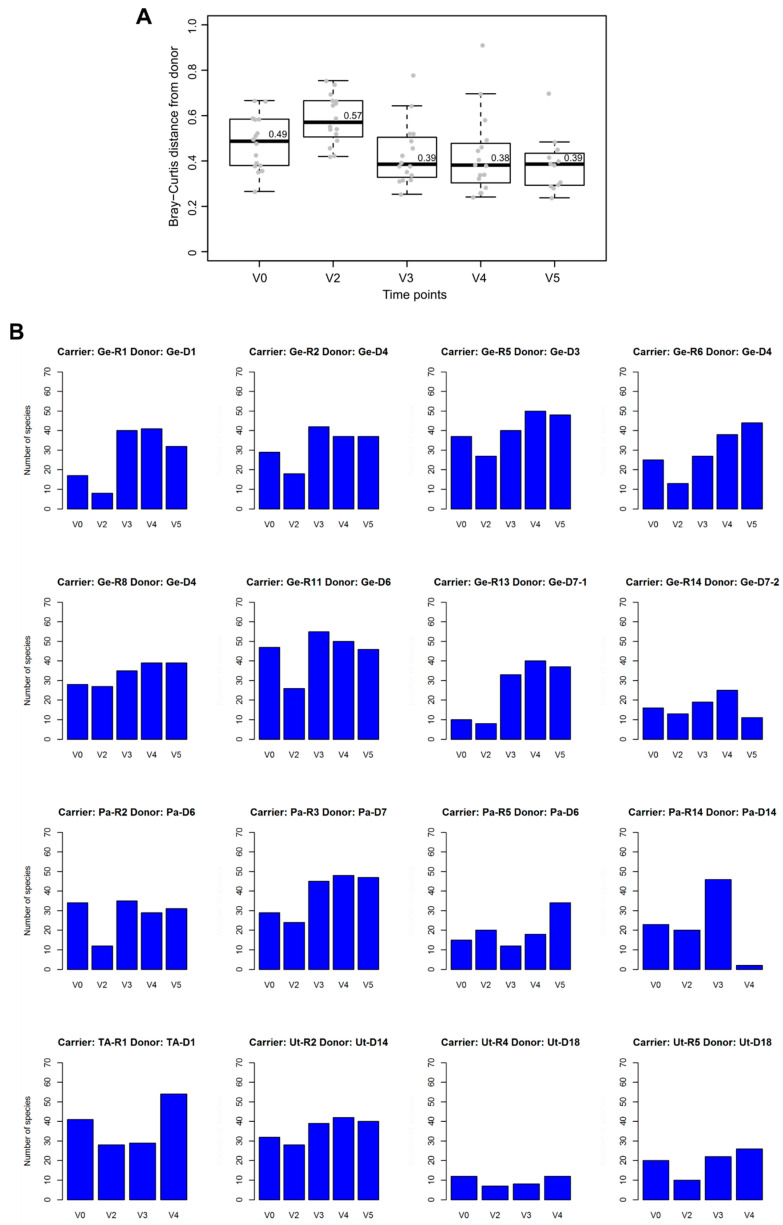
Donor microbiota and their associations with recipient microbiota. (**A**) Boxplot representing the Bray–Curtis distance (see the main text) between donors and FMT-treated carriers at each time point. Median values are reported above the corresponding (thick) line of boxplots. (**B**) Bar plots representing the number of species shared with the donor at each time point for each FMT-treated carrier. Carrier and corresponding donor identifiers are indicated at the top of each bar plot.

To analyze the association between stool microbiota of carriers and donors, we computed the Bray–Curtis index (BCi), where the higher the value, the more similarity there is between the two groups. BCi median values for donor versus carrier microbiota were lower at post-FMT time points V3 and V4 than at V0 and at V2; however, no significant p-values were associated with these differences (Figure 3A). 

**Figure 4:**
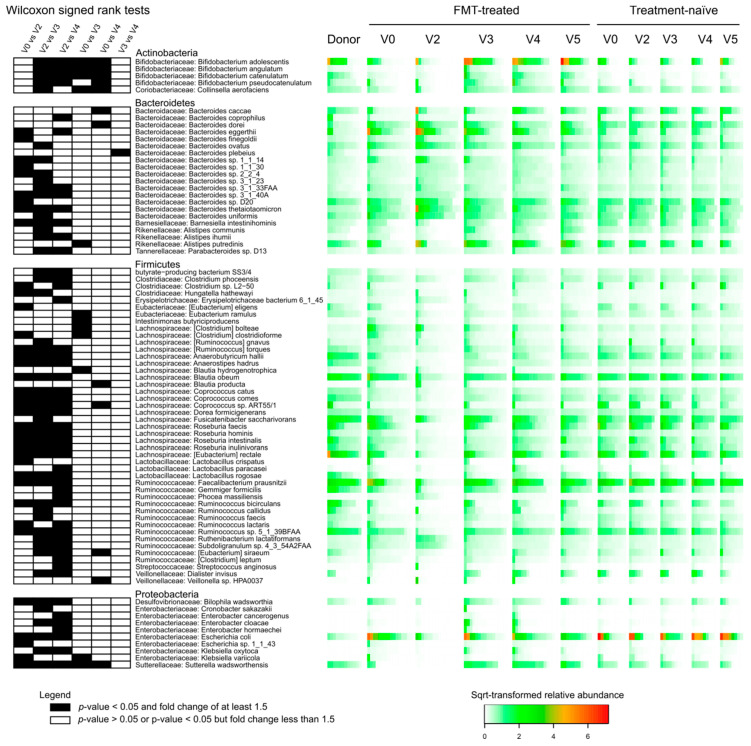
Differentially abundant species in FMT-treated carriers. For each of the 81 represented species, we report the statistical significance (Wilcoxon signed rank test) of changes in the relative abundance between time points (left), phylum and family assignments (middle) and the relative abundance in donor, FMT-treated and treatment-naïve carriers (right). Importantly, we selected species for which at least one of the 6 pairwise comparisons reported (V0 vs. V2, V0 vs. V3, V0 vs. V4, V2 vs. V3, V2 vs. V4, V3 vs. V4) was statistically significant (*p* < 0.05) with a ≥1.5 fold change in the relative abundance and a mean relative abundance ≥0.1% in at least one of the two compared groups. Significant differences (*p* < 0.05) associated with a ≥1.5 fold change in the relative abundance are represented as black shaded cells; white cells denote other cases. Relative abundances are square-root-transformed and color-scaled as indicated in the legend.

For each of the 16 FMT-treated carriers, we identified species shared with their corresponding donors. In most cases, the number of these shared species was higher at V3 and V4 than at baseline and V2 (Figure 3B). Of 32 species that were shared with the donor at V3 or V4 in at least half (n ≥ 8) transplanted carriers (Table 1), 23 were found to be differentially abundant (Wilcoxon signed rank test, *p* < 0.05) in at least one pairwise comparison between the first four time point samples (V0, V2, V3 and V4) of FMT-treated carriers (Figure 4). 

We found that four *Bifidobacterium* species and *Collinsella aerofaciens* (Actinobacteria) were significantly more abundant in post-FMT time points than at V0 and V2 (Figure 4). Several other species, belonging to the genera *Bacteroides*, *Alistipes* (Bacteroidetes), *Eubacterium*, *Clostridium*, *Intestinimonas*, *Blautia*, *Coprococcus*, *Veillonella* (Firmicutes), *Klebsiella* and *Sutterella* (Proteobacteria), were also found differentially abundant between baseline and post-FMT time points (Figure 4). The antibiotic treatment was associated with significant changes in the relative abundance between V2 and all three other time points (V0, V3 and V4) for 23 species from the phyla Bacteroidetes, Firmicutes and Proteobacteria (Figure 4). 

### 3.4. Comparisons of Microbiota of FMT-Treated and Treatment-Naïve Individuals

Differences in microbiota composition between the time points in treatment-naïve carriers were not statistically significant (PERMANOVA, *p* > 0.05). Likewise, we did not detect significant differences in global microbiota composition and ecological indexes between FMT-treated and treatment-naïve carriers except at the time point V2. *Bifidobacterium* species, including *B. adolescentis*, *B. angulatum*, *B. catenulatum*, *B. pseudocatenulatum*, were significantly more abundant in FMT-treated than in treatment-naïve carriers at V4 and/or V3 (post-FMT) time points (Wilcoxon rank sum test, *p* < 0.05; Figure S2). On the other hand, *Lactobacillus ruminis* had significantly lower relative abundance at V3, V4 and V5 in FMT-treated subjects when compared to treatment-naïve individuals (Wilcoxon rank sum test, *p* < 0.05; Figure S2).

### 3.5. Effect of FMT/antibiotics on the Abundance of Proteobacteria, Enterobacteriaceae and of Selected Beta-lactam ARDs

**Figure 5:**
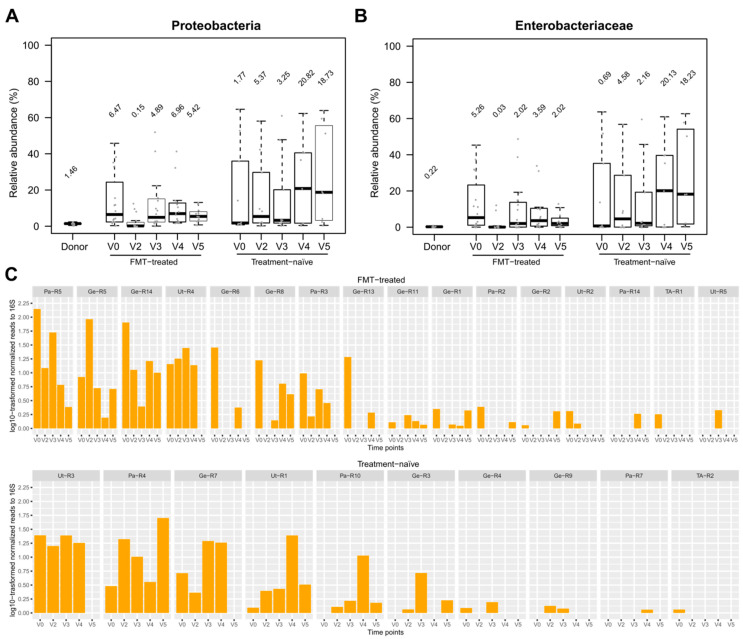
Effect of antibiotics/FMT treatment on the relative abundance of Enterobacteriaceae and the abundance of putative ESBL and carbapenemase genes. Boxplots combined with dotplots represent the relative abundance of Proteobacteria (**A**) and Enterobacteriaceae (**B**) in donors, FMT-treated and treatment-naïve carriers. (**C**) Counts of reads assigned to detected *bla*_CTX-M_, *bla*_NDM_, *bla*_OXA_, *bla*_SHV_ and *bla*_TEM_ genes were normalized to read counts of 16S rRNA genes and multiplied by 1000 prior to log10-transformation.

Proteobacteria and Enterobacteriaceae levels were higher in carriers as compared to donors (Figure 5). In FMT-treated carriers, colistin/neomycin treatment resulted in a transient and significant decrease in the abundance of Proteobacteria and Enterobacteriaceae (Wilcoxon signed rank test, *p* < 0.05; Figure 5A,B). After FMT administration, the abundance of Enterobacteriaceae remained lower relative to baseline but without statistical significance. *Escherichia coli*, the most abundant Enterobacteriaceae species detected in our analyses, followed a similar temporal pattern (Figure 4). 

We assessed the abundance of putative *bla*_CTX-M_, *bla*_NDM_, *bla*_OXA_, *bla*_SHV_, *bla*_TEM_ and *bla*_KPC_ genes (from the ResFinder database), which may be associated with ESBL-E and CPE [17,18], to determine whether antibiotics/FMT treatment caused a change in the abundance of these resistance genes. This analysis included samples from 14 FMT-treated individuals who had detectable *bla* genes (*bla*_CTX-M_, *bla*_NDM_, *bla*_OXA_, *bla*_SHV_ and *bla*_TEM_) at baseline. In 10 of them, the *bla* gene content was higher at baseline than at later time points (Figure 5C). Importantly, no such temporal pattern was observed in treatment-naïve carriers (Figure 5C). 

### 3.6. Comparison of Metagenomic Results with R-GNOSIS ESBL-E/CPE Decolonization Outcomes

The primary objective of the R-GNOSIS WP3 trial was to evaluate if antibiotics followed by FMT treatment resulted in the absence of detectable carriage of ESBL-E/CPE at V4 by analyzing stool culture at this time point. We therefore subdivided FMT-treated and treatment-naïve carriers in “decolonized” and “persistently colonized” categories depending on whether their fecal samples were found ESBL-E/CPE negative or positive at V4 in the R-GNOSIS study. Accordingly, 9 (out of 16) FMT-treated carriers and three (out of 9) treatment-naïve carriers were considered decolonized (Table S1). The treatment-naïve carrier TA-R2 was excluded from this analysis as no sample for this subject was taken at V4.

Colistin/neomycin administration had a significant impact on microbiota composition irrespective of the V4-decolonization status; in PCoA, fecal microbiota from V2 clustered apart from the other time point samples (Figure 1B and Figure S3A), and these differences were significant (PERMANOVA, *p* < 0.05) in both persistently colonized and decolonized subgroups. Microbial profiles of FMT-treated carriers were significantly different between decolonized and persistently colonized groups (PERMANOVA test, *p* < 0.05) at V3 but not at V0, V2, V4 and V5. For treatment-naïve carriers (Figure S3B), differences in microbiota between decolonized and persistently colonized carriers were not statistically significant (PERMANOVA, *p* > 0.05) at any time point.

Eventually, we compared metagenomic predictions of the presence of detected ESBL and carbapenemase genes (*bla*_CTX-M_, *bla*_NDM_, *bla*_OXA_, *bla*_SHV_ and *bla*_TEM_) with the results from R-GNOSIS WP3 conventional testing. Phenotypic analyses did not consistently match with metagenomic predictions of ESBL-E/CPE carriage (Figure S3C). Decolonization status (ESBL-E/CPE negative or positive) at V4 had concordant outcomes between the two approaches (phenotypic and metagenomic) in 68% (17/25) of cases (77.8% [7/9] for treatment-naïve and 62.5% [10/16] for FMT-treated carriers; see also Table S2).

## 4. Discussion

The R-GNOSIS WP3 study [11] was the first multicenter randomized clinical trial systematically investigating FMT for decolonization of ESBL-E/CPE from the gut. In the present study we characterized the recipient and donor fecal microbiota of a sub-cohort of the R-GNOSIS WP3 participants by whole metagenome shotgun sequencing. 

The first main finding of our analyses was that antibiotic treatment resulted in a profound change in microbiota composition with reduced species richness and diversity, lower Firmicutes/Bacteroidetes ratio and decreased proportions of Proteobacteria and Enterobacteriaceae. A decreased Firmicutes/Bacteroidetes ratio has been previously reported in hypertensive rat models following neomycin treatment [19]. 

Metagenomic predictions for ESBL-E and CPE were 72% concordant with phenotypic screening and both approaches have their own advantages and disadvantages. In line with the results of phenotypic antibiotic susceptibility tests performed in the main study [11], we did not detect acquired colistin resistance genes, although we cannot exclude the presence of resistance mechanisms not detectable by our approach [20]. Determinants encoding resistance to aminoglycosides, a class to which neomycin belongs, were abundant in our metagenomic data, even in samples collected before treatment. Since phenotypic neomycin susceptibility testing was not performed in the main study, the correlation between metagenomic and culture-based results could not be assessed. The most frequently identified ARDs in our study were tetracycline resistance genes, which have been previously shown to be the most abundant ARDs in human gut commensals but with a marked geographic variation [21,22]. A large use of aminoglycosides, tetracycline and other antibiotics in animal rearing probably promoted transmission of ARDs from food animals to humans [22]. 

Effects of antibiotic treatment on the species richness and diversity as well as on ARD content were transitory; after FMT administration, their values restored to baseline levels. In particular, the gut microbiota from post-FMT time points, as compared to V2 (the end of antibiotic treatment), was significantly enriched in Lachnospiraceae (Firmicutes), Ruminococcaceae (Firmicutes), and *Alistipes* species (Bacteroidetes). These taxa include producers of propionate and butyrate [23], which are known to reduce inflammation of intestinal mucosa [24].

Relative to the baseline, post-FMT microbiota was significantly enriched in *Bifidobacterium* species and *C. aerofaciens*. No such increase was detected over the same time period in treatment-naïve carriers. *Bifidobacterium* species are known to attenuate inflammation [25] and some strains of *B. pseudocatenulatum* and *B. catenulatum* recovered from human fecal samples have been shown to exert growth inhibition on enterotoxigenic *E. coli* strain cultures [26]. *C. aerofaciens* might have an anti-inflammatory effect on intestinal epithelium since this species also includes butyrate-producer strains [27]. 

In FMT-treated carriers, we found a decrease in the proportion of Enterobacteriaceae in post-FMT-microbiota compared to baseline, albeit not statistically significant, and we observed that putative ESBL and carbapenemase genes were more abundant at baseline than at any later sampling point in 10 out of 16 cases. 

In a nonrandomized prospective single-center cohort study conducted on seven hematologic patients in Poland, higher abundance of *Barnesiella intestini hominis* in donor fecal microbiota was associated with successful decolonization of *Klebsiella pneumoniae* harboring New Delhi metallo-β-lactamase-1 (NDM-1) in FMT recipients [28]. Moreover, the genus *Barnesiella* has been reported to confer resilience to vancomycin-resistant *Enterococcus faecium* intestinal colonization in a mouse model [29]. In our study, the relative abundance of *Barnesiella intestini hominis* significantly increased after FMT-treatment (V3) as compared to V2 (Figure 4).

Although the overall (Bray–Curtis) dissimilarity between donor and carrier microbiota was not significantly different for baseline and post-FMT time points, our results suggest that FMT increases the proportion of species antagonistic to ESBL-E and of species that reinforce the gut epithelium barrier by modulating inflammatory processes. 

Microbiota collected at V3 (after FMT), but not at the next visit (V4), at which the decolonization status was defined, were significantly different between colonized and decolonized individuals, which suggests that gut decolonization of ESBL-E/CPE was associated with short-term microbiota changes.

Our study has several limitations: (i) the sample size of our cohort makes it difficult to reach definitive conclusions; (ii) the limited number of donors did not allow us to discriminate possible differences in preservation of donor microbiota components between capsules and suspensions for nasogastric tube administration; (iii) in analyses aimed at identifying differentially abundant species, we excluded V5 samples because several carriers had not been sampled at this time point; (iv) metagenomic reads assigned to ARDs, fungi and viruses had low counts. Nevertheless, in line with previous studies [30,31,32], DNA bacteriophages (*Myoviridae*, *Podoviridae* and *Siphoviridae* families) were the most abundant viruses and *Saccharomyces* was the most frequently identified fungal genus (Figure S4).

In conclusion, our findings give insights on microbiota composition under combined treatment of antibiotics and FMT. Following this treatment, increased relative abundance of certain species possibly limits the colonization by ESBL-E/CPE. Further studies with larger sample size and use of FMT without antibiotics are needed to clarify these observations.

## Figures and Tables

**Table 1:** Thirty-two species most frequently shared between the donors and the transplanted carriers. Species present in at least 8 FMT-treated carriers at V3 or at V4 are shown. For each species, numbers indicate in how many carriers they were present at a given visit. For each visit, we report the number of FMT-treated carriers analyzed (“n”). Species reported in Figure 4 are marked with an “X” in the column “Differentially abundant”.

Species	V0 (n = 16)	V2 (n = 16)	V3 (n = 16)	V4 (n = 16)	V5 (n = 12)	Differentially Abundant
*Alistipes putredinis*	4	3	9	9	7	X
*Alistipes shahii*	5	6	8	7	6	
*Anaerostipes hadrus*	6	1	9	9	7	X
*Bacteroides caccae*	4	4	7	11	9	X
*Bacteroides ovatus*	11	12	13	13	10	X
*Bacteroides* sp. 4_1_36	10	11	11	11	8	
*Bacteroides* sp. D20	12	13	14	13	10	X
*Bacteroides stercoris*	6	5	10	10	8	
*Bacteroides thetaiotaomicron*	9	10	11	10	7	X
*Bacteroides uniformis*	13	14	12	13	11	X
*Bacteroides vulgatus*	13	14	11	12	10	
*Bifidobacterium adolescentis*	4	1	8	9	8	X
*Bifidobacterium catenulatum*	2	0	8	7	5	X
*Bifidobacterium longum*	13	9	15	14	11	
*Blautia obeum*	14	9	15	15	11	X
*Collinsella aerofaciens*	4	2	14	13	10	X
*Coprococcus comes*	5	2	8	10	8	X
*Dorea formicigenerans*	7	3	11	11	8	X
*Dorea longicatena*	8	3	11	12	11	
*Escherichia coli*	9	3	6	9	8	X
*[Eubacterium] rectale*	7	4	10	11	10	X
*Faecalibacterium prausnitzii*	12	4	13	11	11	X
*Fusicatenibacter saccharivorans*	9	7	12	12	10	X
*Gemmiger formicilis*	6	2	12	12	11	X
Lachnospiraceae bacterium 7_1_58FAA	8	5	7	8	5	
*Odoribacter splanchnicus*	9	7	8	10	6	
*Roseburia faecis*	11	0	9	10	9	X
*Roseburia intestinalis*	12	1	7	12	9	X
*Roseburia inulinivorans*	9	0	7	10	8	X
*Ruminococcus* sp. 5_1_39BFAA	13	1	15	15	11	X
*Ruminococcus* sp. SR1/5	11	6	12	13	10	
*Sutterella wadsworthensis*	3	0	11	11	7	X

## Supplementary Materials

The following are available online at https://www.mdpi.com/2076-2607/8/6/941/s1, Figure S1: ARD classes, Figure S2: Differentially abundant species between FMT-treated and treatment-naïve carriers, Figure S3: Comparisons of metagenomic analyses with R-GNOSIS ESBL-E/CPE decolonization outcomes, Figure S4: Most abundant viral families and fungal genera detected by metagenomics, Table S1: Summary table describing gender, age and body mass index (BMI) of donors and carriers analyzed in this study, Table S2: Comparison of ESBL-E/CPE detection as performed by phenotypic tests and as predicted by metagenomic analyses for the sub-cohort of ESBL-E/CPE carriers analyzed in the present study.
