# Supplementary material for: Metagenomic Characterization of Gut Microbiota of Carriers of Extended-Spectrum Beta-Lactamase or Carbapenemase-Producing Enterobacteriaceae Following Treatment with Oral Antibiotics and Fecal Microbiota Transplantation: Results from a Multicenter Randomized Trial
Source: Microorganisms. 2020 Jun 23;8(6):941. doi: 10.3390/microorganisms8060941 (PMC7357103; doi:10.3390/microorganisms8060941)
Supplement: Supplementary file 1 [file microorganisms-08-00941-s001.pdf]

Supplementary Materials

Metagenomic Characterization of Gut Microbiota of Carriers of Extended-Spectrum Beta-Lactamase or Carbapenemase-Producing Enterobacteriaceae Following Treatment with Oral Antibiotics and Fecal Microbiota Transplantation: Results from a Multicenter Randomized Trial

Stefano Leo, Vladimir Lazarevic, Myriam Girard, Nadia Gaïa, Jacques Schrenzel, Victoire de Lastours, Bruno Fantin, Marc Bonten, Yehuda Carmeli, Emilie Rondinaud, Stephan Harbarth and Benedikt D. Huttner.

Supplementary Methods

Additional information on the sub-cohort chosen for the present study

Sixteen out of 26 ESBL-E/CPE carriers were FMT-treated (Ge n = 8, Pa n = 4, Ut n = 3, TA n = 1). The 10 other carriers (Ge n = 4, Pa n = 3, Ut n = 2, TA n = 1) were from the treatment-naïve group. Seven corresponding stool donors (1 from TA and Ut, 2 from Pa and 3 from Ge) were also selected.

For four individuals of the FMT-treated group (Pa-R14, TA-R1, Ut-R4 and Ut-R5) and two from the treatment-naïve group (Ut-R3 and TA-R2), the sample from time point V5 was missing. Feces were also not sampled from TA-R2 at V4.

For donors, we sequenced DNA from aliquots of native feces and/or of FMT preparation.

Antibiotic treatment and fecal microbiota transplantation

Antibiotic treatment consisted of administration of colistin sulphate (2 million international units 4×/day per os) and neomycin sulphate tablets (350 mg of neomycin base 4×/day per os) for 5 days. On the second calendar day after the end of antibiotic treatment, FMT was performed without prior bowel lavage. Two centers (Ge, Pa) used oral, capsulized FMT (15 capsules on two consecutive days), the other two centers (Ut, TA) performed FMT via nasogastric tube (80 mL of FMT preparation on a single day).

Preparation and storage of samples from ESBL-E/CPE carriers

Stools from carriers were collected at the study sites or by the patients at home, conserved at room temperature and transported to the microbiology lab in maximum 72 hours from their emission. Feces were homogenized with a BagMixer 400P (Interscience, SaintNom-la-Bretèche, France), 1/10 diluted in saline and frozen in 500-µL aliquots at –80°C.

Donor sample preparation and storage

Fecal suspensions for nasogastric tube procedure were prepared as follows: homogenized stool was suspended in sterile non-bacteriostatic normal saline (5 mL per g of stool), filtered and centrifuged. Fecal concentrate was suspended in one-third the original volume in saline containing 10% glycerol to obtain a final volume of about 80 mL per 40 g of stool. For capsule preparation, fecal concentrate, obtained from the initial stool suspension (6 mL saline per g of stool), was suspended in one-tenth the original volume in saline containing 80% glycerol. Both types of fecal suspensions and native stools were kept frozen at –80 °C. For details see Huttner et al. 2019 [1].

DNA extraction from stool samples and sequencing

DNA extraction was performed as described by Frossard et al. [2] with some modifications. Briefly, 200 µL of frozen fecal suspension or native stools and 400 µL Buffer GT (RBC Bioscience, New Taipei City, Taiwan) were added to a Nucleospin Bead Tube containing ceramic beads (Macherey-Nagel, Düren, Germany). After shaking for 20 min at maximum speed on a Vortex-Genie 2, RNA was removed by incubating the mixture for 5 min with 1 µL of 50 mg/mL RNAse A (Roche, Basel, Switzerland). The lysate was then centrifuged for 1 min at 11,000 × g and 400 µL of supernatant loaded into a MagCore Sample Tube of a MagCore Genomic DNA Tissue Kit (RBC Bioscience). DNA extraction was then performed on a MagCore HF16 Automated Nucleic Acid Extractor (RBC Bioscience) using the program 401. DNA was eluted in 100 µL of Tris 10 mM pH8. The concentration of DNA was measured by QuBit dsDNA BR Assay Kit (Life Technologies, Carlsbad, CA, USA). DNA libraries were prepared with NextSeq 500 High-Output v2 kit (Illumina, San Diego, CA, USA) and sequenced (2 × 150) on a NextSeq 500 (Illumina) at LGC Genomics GmbH (Berlin, Germany). In total, we sequenced 152 samples: 21 from 7 donors, 76 from the 16 FMT-treated carriers, 47 from the 10 treatment-naïve carriers and 8 negative extraction controls (DNA extracts obtained by adding no sample).

Pre-processing, taxonomic classification and relative abundance computation

The quality of raw reads was inspected with the FastQC tool (http://www.bioinformatics.babraham.ac.uk/projects/fastqc/). Raw reads were adapter-trimmed and scanned for quality filtering with Trimmomatic v0.36 [3] (sliding window size = 20, average quality = 28, minimum length = 100). Duplicated reads were removed with in-house perl scripts and those mapping by Kraken2 [4] to human genome (GRCh38.p7) were removed. The remaining reads were then assigned to bacteria, viruses and fungi by Kraken2 using a confidence score of 80%. The reference genome database was constructed as described by Kirstahler [5]. We added to the reference database 367 genomes available (as of September 2018) from Gastrointestinal Human Microbiome Project (GI HMP) (The Human Microbiome Project [6]). In total, 13,537 genomes were used: 5949 from bacteria, 7532 from viruses and 56 from fungi. The relative abundance of reads mapping to a given bacterial species was expressed as a percentage of the total number of reads assigned to bacterial phyla. The relative abundance of viral families and fungal genera was computed by taking into account the total number of microbial (bacterial, viral and fungal) reads.

Sequencing data availability

Quality-filtered dereplicated sequencing reads were deposited as FASTQ files at the European Nucleotide Archive (ENA) under the project PRJEB35816. Reads assigned to human genome (GRCh38.p7) by Kraken2 were removed before data submission.

Identification of antibiotics resistance determinants and of 16S rRNA genes

After removing sequences assigned to human genome by Kraken2, dereplicated R1 reads were mapped against the ResFinder database [7] with USEARCH (usearch_global -id 0.9 -strand both -top_hits_only -mincols 100) to detect antibiotics resistance determinants (ARDs).

Dereplicated host-depleted R1 reads with minimal length of 120 nt were also mapped against EzBioCloud 16S rRNA gene sequence database [8] with USEARCH v10 [9] (-id 0.97 -query_cov 1 -top_hit_only -strand both).

For sequences with multiple best hits to ARDs, we kept the hit corresponding to the reference sequence with the highest number of matches for the entire dataset. To compute the ARD content of a sample, the sum of read counts mapping to a ResFinder gene or antimicrobial class was divided by the

sum of reads mapping to the 16S rRNA gene. The obtained ratios were multiplied by 1000. Prediction of ESBL-E/CPE carriage was performed by querying the ResFinder databse for bla_{CTX-M}, bla_{NDM}, bla_{OXA}, bla_{SHV}, bla_{TEM} and bla_{KPC} genes.

Filtering of bacterial species

First, we filtered out the reads corresponding to bacterial species that had higher relative abundance in negative extraction controls (n = 8) than in test samples (n = 144). Those species were likely to result from contaminants present in DNA extraction reagents or from sample handling. We then removed species with a mean relative abundance < 0.001%, resulting in a dataset containing 402 species.

Statistical analyses

Sample selection. For donors, we only analyzed samples that were used for FMT transplantation (via oral capsule or nasogastric route).

Principal coordinates analysis. Principal coordinates analysis was performed in R v3.2.3 using vegan v2.3-5 package [10]. Bray–Curtis index was computed with vegdist (vegan) after square-root transformation of species relative abundance. Centriods, centroids coordinates and samples coordinates were computed with betadisper (vegan).

Pairwise comparisons of global microbiota profiles. Bacterial communities were tested for similarities and differences in relation to predictor variables with permutational analysis of variance (pairwise PERMANOVA, with 9999 permutations, unrestricted permutation of raw data and Type III sums of squares) based on the Bray–Curtis similarity matrices of square-root transformed species relative abundance. PERMANOVA tests were performed in PRIMER v7 (PRIMER-E Ltd, Plymouth, UK).

The feces of one carrier (TA-R2) was not collected at V4 and therefore all samples from this individual were excluded when comparing decolonized and persistently colonized groups. TA-R2, initially assigned to intervention group after randomization, received no treatments; this subject was therefore re-assigned to the treatment-naïve group.

Ecological indexes. Before alpha-diversity analyses (species diversity and richness), the counts of reads classified at the species level were rarefied to 40,000 with the rarefy function in R (vegan package). Shannon diversity was computed with the R diversity function (vegan). For calculating richness, a species was considered present in a sample if represented by at least one read.

Identification of differentially abundant species. We performed two-sided Wilcoxon signed rank to evaluate the significance of intra-individual changes of species abundance between different time points, otherwise two-sided Wilcoxon rank sum test was used. All tests were done in R software.

Species shared with the donor

To compute the number of species shared between an FMT-treated carrier of ESBL-E/CPE at a given time point and a corresponding donor, we selected species with a relative abundance > 0.1% in both the carrier and the donor. Species shared between carriers and donors were identified by using the venn function implemented in R package gplots v3.0.1. We then determined the frequency of each shared

species over the total number of patients analyzed at each time point. Of note, at V5, stools were collected from 12 out of 16 FMT-treated carriers.

Supplementary Figures

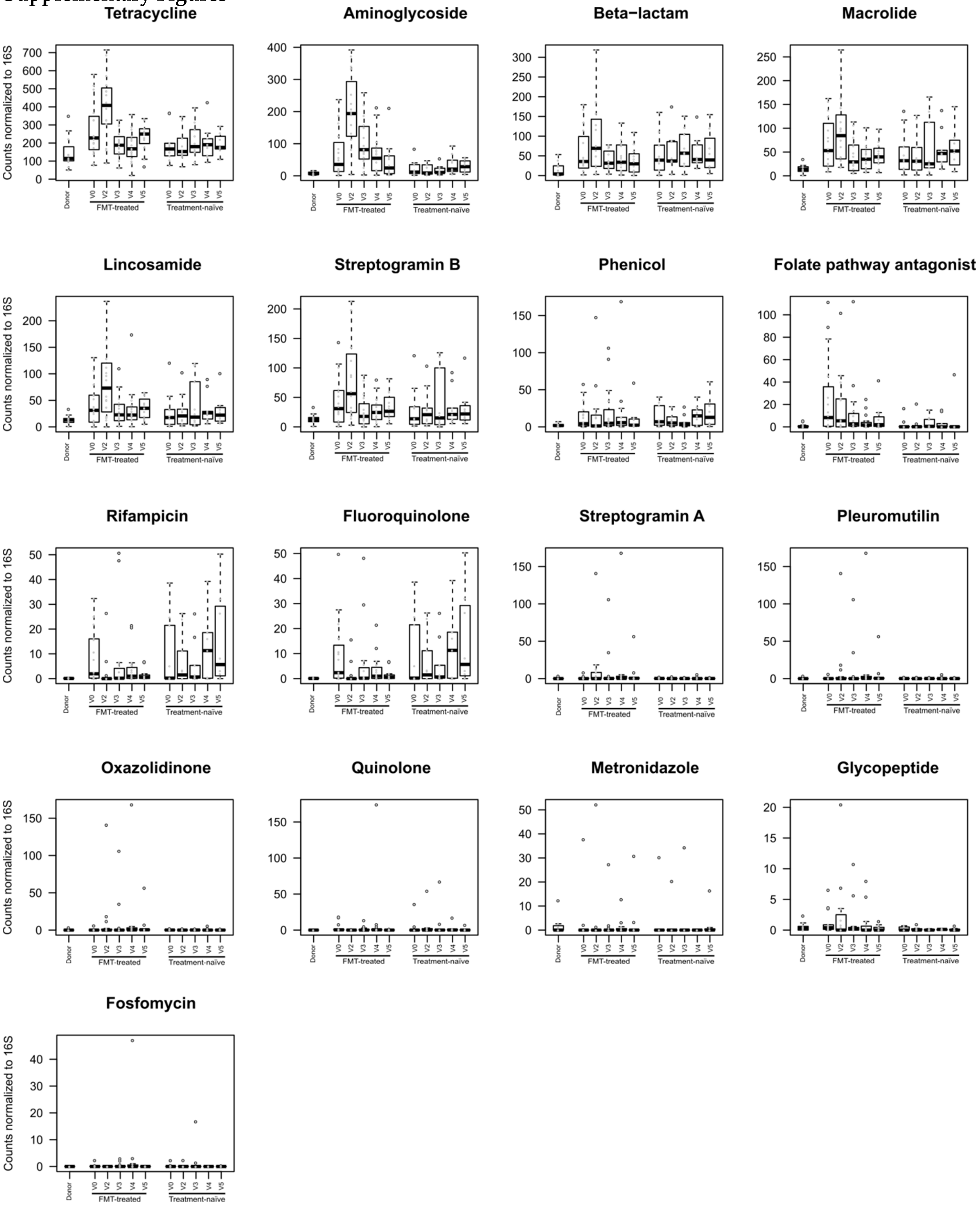

Figure S1. The abundance of ARDs from different anitimicrobial classes. Boxplots report the counts of reads assigned to a given ARD class normalized to 1000 16S rRNA gene reads counts.

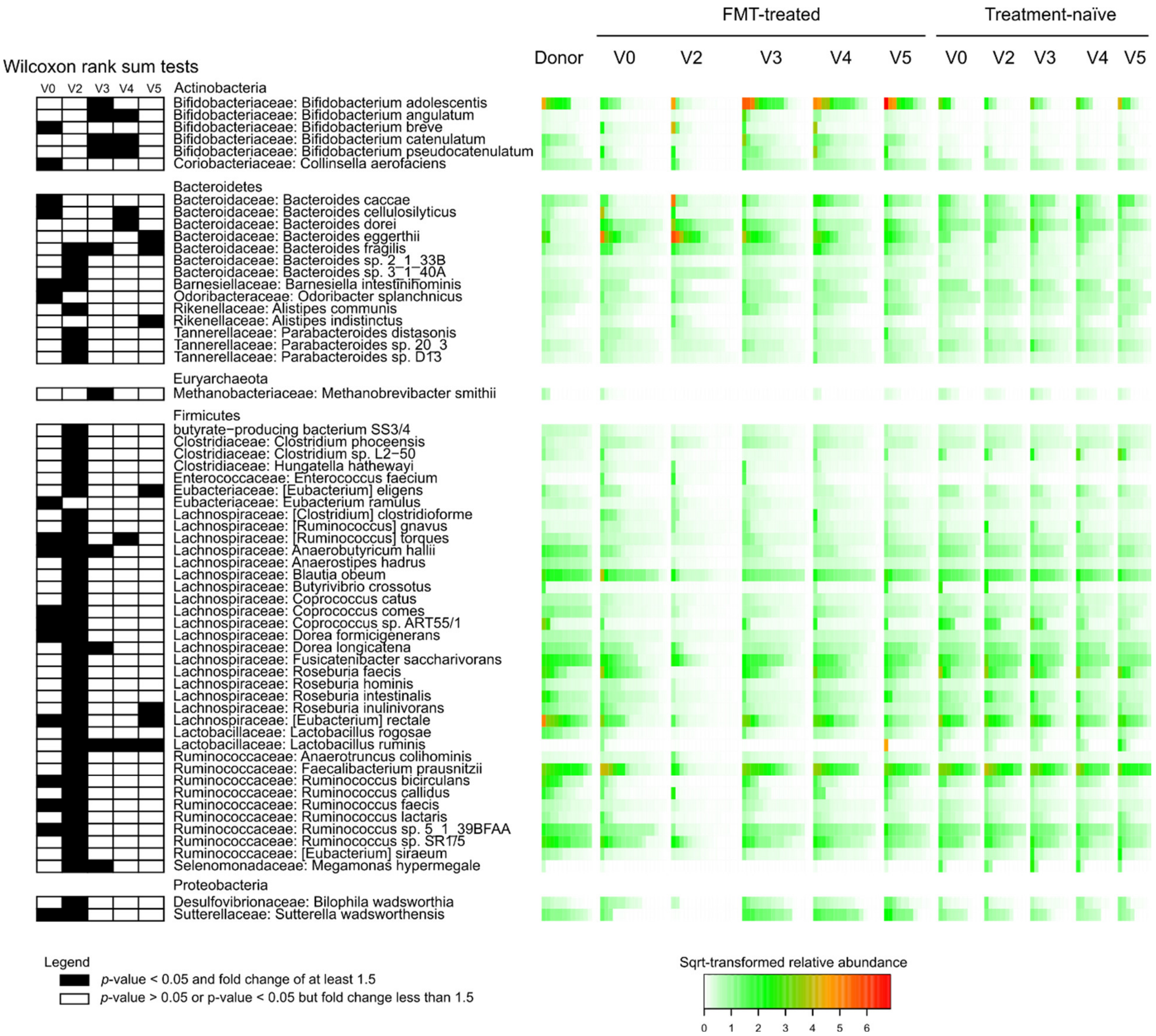

Figure S2. Differentially abundant species between FMT-treated and treatment-naïve carriers. For each of the 60 selected species, we report the corresponding phylum and family (middle), the significance of differences in the relative abundance between FMT-treated and treatment-naïve carriers at a given time point (left) and the relative abundance in donor, FMT-treated and treatment-naïve carriers (right). We selected species associated with a significant Wilcoxon rank sum test ($p < 0.05$), a fold change ≥ 1.5 in the relative abundance and a mean relative abundance ≥ 0.1% in at least one of the compared groups. Significant differences ($p < 0.05$) associated with a ≥ 1.5 fold change in the relative abundance are represented as black shaded cells; white cells denote other cases. Relative abundances are square-root-transformed and color-scaled as indicated in the legend.

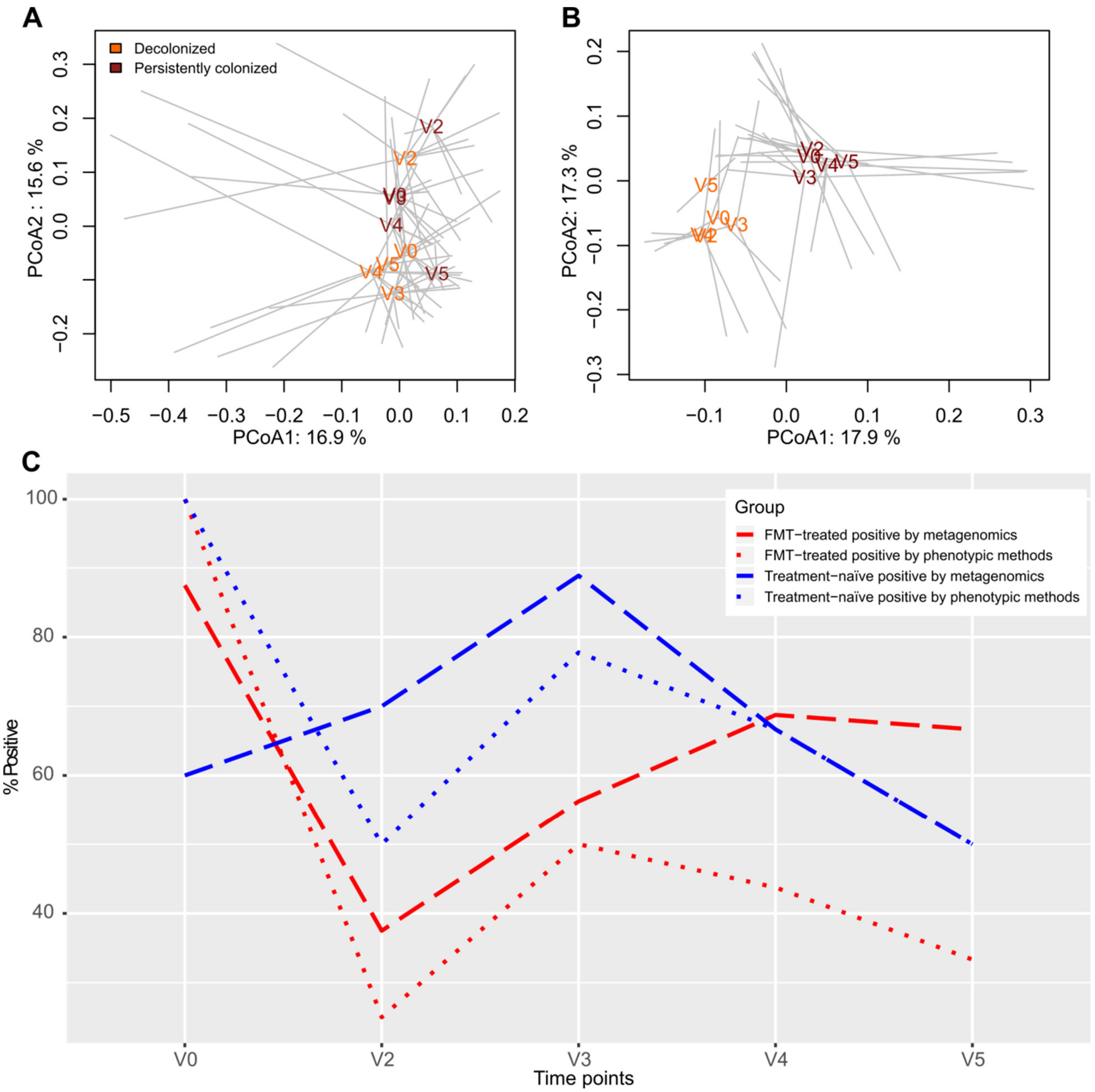

Figure S3. Differences in microbial communites between ESBL-E/CPE persistently colonized and decolonized carriers and comparisons of metagenomic predictions with culture-based (R-GNOSIS study) ESBL-E/CPE decolonization outcomes. PCoA plots of microbiota profiles computed on species relative abundance of FMT-treated (**A**) and treatment-naïve (**B**) carriers. Colors are according to the V4-decolonization status (determined in the R-GNOSIS study). (**C**) ESBL-E/CPE positivity detected by phenotypic tests (R-GNOSIS study) and predicted by metagenomic analyses (this study). The percentage of carriers detected positive at the five analyzed time points are reported. TA-R2 was excluded from the analyses reported in panels (**B**) and (**C**).

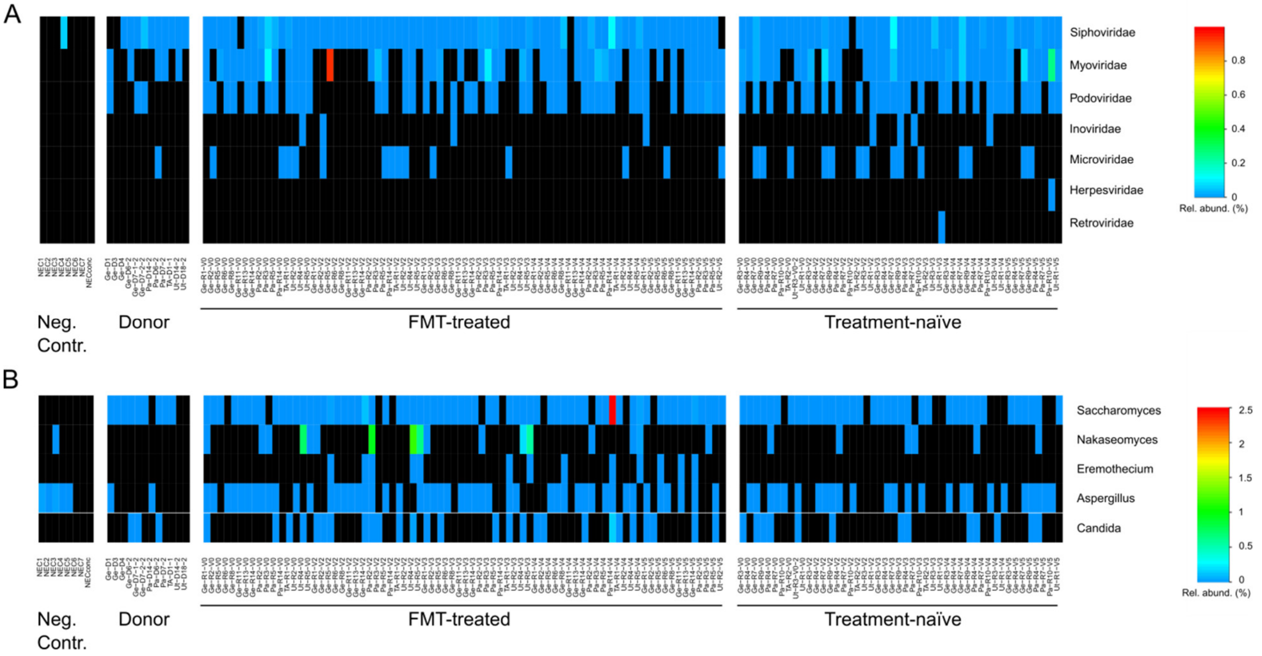

Figure S4. Most abundant viral families and fungal genera detected by metagenomics. (**A**) Heat map showing the relative abundance of viral families per sample. (**B**) Per-sample relative abundance of the 5 most abundant fungal genera detected. Black cells indicate undetected taxa.

Supplementary Tables

Table S1. Summary table describing gender, age and body mass index (BMI) of donors and carriers analyzed in this study.

	**FMT-treated**	**Treatment-naïve**	**Donor**
**Total**	16	10	7
**Sex, male**	8	5	5
**Age, years***	64 [17]	64 [9]	29 [10]
18–34	2	0	6
35–56	0	0	1
57–64	5	6	0
64	9	4	0
**BMI, kg/m²***	26.2 [4.9]	31.7 [7.3]	
< 18.5 (Underweight)	0	0	
18.5–24.9 (Normal weight)	8	1	
25.0–29.9 (Pre-obesity)	5	3	
≥ 30.0 (Obesity)	3	6	
Successfully decolonized from ESBL-E/CPE carriage according to R-GNOSIS WP3	9	3	-

* data are: mean [standard deviation]

Table S2. Comparison of ESBL-E/CPE detection as performed by phenotypic tests and as predicted by ARD metagenomics analyses for the sub-cohort of ESBL-E/CPE carriers analyzed in the present study*.

			Phenotypic tests		Concordance (%)
			Negative	Positive	
All carriers (n = 25) all time points	Metagenomics (predictive)	Negative	30	13	72.1
		Positive	21	58	
All carriers at V4	Metagenomics (predictive)	Negative	6	2	68
		Positive	6	11	
Treatment-naïve carriers (n = 9) at V4	Metagenomics (predictive)	Negative	2	1	77.8
		Positive	1	5	
FMT-treated carriers (n = 16) at V4	Metagenomics (predictive)	Negative	4	1	62.5
		Positive	5	6	

* The sample from time point V5 was not available for four individuals of the FMT-treated group (Pa-R14, TA-R1, Ut-R4 and Ut-R5) and two individuals from the treatment-naïve group (Ut-R3 and TA-R2). For TA-R2, stool samples were not analyzed by culture at V3 and not collected at V4.

References

1. Huttner BD, de Lastours V, Wassenberg M, Maharshak N, Mauris A, Galperine T, Zanichelli V, Kapel N, Bellanger A, Olearo F *et al*: A 5-day course of oral antibiotics followed by faecal transplantation to eradicate carriage of multidrug-resistant Enterobacteriaceae: a randomized clinical trial. *Clinical Microbiology and Infection*.
2. Frossard CP, Lazarevic V, Gaïa N, Leo S, Doras C, Habre W, Schrenzel J, Burger D, Eigenmann PA: The farming environment protects mice from allergen-induced skin contact hypersensitivity. *Clinical & Experimental Allergy* **2017**, *47(6)*:805-814.
3. Bolger AM, Lohse M, Usadel B: Trimmomatic: a flexible trimmer for Illumina sequence data. *Bioinformatics* **2014**, *30(15)*:2114-2120.
4. Wood DE, Salzberg SL: Kraken: ultrafast metagenomic sequence classification using exact alignments. *Genome Biology* **2014**, *15(3)*:R46.
5. Kirstahler P, Bjerrum SS, Friis-Møller A, la Cour M, Aarestrup FM, Westh H, Pamp SJ: Genomics-based identification of microorganisms in human ocular body fluid. *Scientific reports* **2018**, *8(1)*:4126-4126.
6. Human Microbiome Jumpstart Reference Strains C, Nelson KE, Weinstock GM, Highlander SK, Worley KC, Creasy HH, Wortman JR, Rusch DB, Mitreva M, Sodergren E *et al*: A catalog of reference genomes from the human microbiome. *Science (New York, NY)* **2010**, *328(5981)*:994-999.
7. Zankari E, Hasman H, Cosentino S, Vestergaard M, Rasmussen S, Lund O, Aarestrup FM, Larsen MV: Identification of acquired antimicrobial resistance genes. *Journal of Antimicrobial Chemotherapy* **2012**, *67(11)*:2640-2644.
8. Yoon S-H, Ha S-M, Kwon S, Lim J, Kim Y, Seo H, Chun J: Introducing EzBioCloud: a taxonomically united database of 16S rRNA gene sequences and whole-genome assemblies. *International journal of systematic and evolutionary microbiology* **2017**, *67(5)*:1613-1617.
9. Edgar RC: Search and clustering orders of magnitude faster than BLAST. *Bioinformatics* **2010**, *26(19)*:2460-2461.
10. Jari Oksanen FGB, Michael Friendly, Roeland Kindt, Pierre Legendre, Dan McGlinn, Peter R. Minchin, R. B., O'Hara GLS, Peter Solymos, M. Henry H. Stevens, Eduard Szoecs and Helene Wagner: vegan: Community Ecology Package. R package version 2.5-6. https://CRAN.R-project.org/package=vegan. **2019**.
